# Optimal Use of Computed Tomography in Diagnosing Internal Herniation After Roux-en-Y Gastric Bypass: A Proposition for the Application of a Radiological Prediction Score

**DOI:** 10.1007/s11695-025-08323-4

**Published:** 2025-10-18

**Authors:** Lilian L. van Hogezand, Lucas Goense, Erik J.R.J. van der Hoeven, Charlotte J. Tutein Nolthenius, Niek van Oorschot, Luigi A.M.J.G. van Riel, Marinus J. Wiezer, Niels A.T. Wijffels, Marijn Takkenberg, Wouter W. Te Riele, Lea M. Dijksman, Hjalmar C. van Santvoort, Wouter J.M. Derksen

**Affiliations:** 1 Department of Surgery, St. Antonius Ziekenhuis, Nieuwegein, Netherlands; 2 Department of Radiology, St. Antonius Ziekenhuis, Nieuwegein, Netherlands; 3 Department of Value Based Health Care, St. Antonius Ziekenhuis, Nieuwegein, Netherlands; 4 Department of Surgery, University Medical Center Utrecht, Utrecht, Netherlands

**Keywords:** Computed tomography (CT), Internal herniation, (Roux-en-Y) gastric bypass, Prediction score

## Abstract

**Background:**

Structured assessment of abdominal computed tomography (CT)-scans is increasingly used to identify signs of internal herniation after Roux-en-Y gastric bypass (RYGB), aiding in the decision-making process to perform a diagnostic laparoscopy (DLS). This study aimed to develop a prediction score based on structured assessment of CT-signs for internal herniation.

**Methods:**

Patients presenting with abdominal pain after RYGB, who underwent a CT-scan for suspicion of internal herniation and subsequently DLS, were included. CT-scans were reassessed for presence of ten CT-signs for internal herniation by two radiologists and two registrars. Diagnostic accuracy for detection of internal herniation for each sign and an overall suspicion score were calculated and compared with the original CT-reports. Interobserver agreement was measured using Fleiss’ kappa. A prediction score was developed based on variables identified by multivariable logistic regression.

**Results:**

With DLS 44 internal herniations (114 CT-scans, 92 patients) were identified. Structured assessment improved diagnostic accuracy compared to the original CT-report (AUC of 0.69 to 0.79, *p* = 0.03), and the positive (67% to 81%) and negative predictive value (75% to 82%). The three-sign prediction score (venous congestion, swirl sign, right-sided anastomosis) resulted in improved diagnostic accuracy compared to the original CT-report (AUC of 0.69 to 0.79, *p* = 0.038). Interobserver agreement of these signs was adequate between all readers (*K* = 0.56–0.75).

**Conclusions:**

Structured assessment of CT-scans improves diagnostic accuracy for internal herniation after RYGB. Our three-sign prediction-model offers a simplified, reproducible alternative to extensive assessment, without compromising the improved diagnostic effectiveness.

**Supplementary Information:**

The online version contains supplementary material available at 10.1007/s11695-025-08323-4.

## Introduction

Internal herniation is one of the most common causes of abdominal pain after Roux-en-Y gastric bypass (RYGB) for morbid obesity. Reported incidences range from 1 to 12%, influenced by the surgical approach and follow-up time [1, 2]. Internal herniation can lead to critical complications such as intestinal ischemia, which can result in sepsis and even death in 1–2% of patients [3]. Prompt and adequate diagnosis is therefore essential.

Diagnosing internal herniation based solely on clinical assessment is difficult, as patients present with varying symptoms and no discriminating abdominal pain profile has been identified [4, 5]. Diagnostic laparoscopy serves as the current gold standard for diagnosing internal herniation and is performed in up to 47% of patients presenting with abdominal pain [6, 7]. However, internal herniation is not confirmed in 28–53% of patients during diagnostic laparoscopy, exposing patients to invasive and expensive procedures in vain [7–10].

Abdominal CT-scanning is used as a noninvasive diagnostic tool to guide the decision for diagnostic laparoscopy. Free text reporting (reporting in a non-structured textual manner) of the CT-scan is commonly used, but false positive and false negative rates as low as 57% and 33%, respectively, have been previously reported [3, 11, 12]. This leads to surgeons basing their decision to perform diagnostic laparoscopy on clinical parameters in around 20% of patients [6, 8].

Recent studies demonstrate that assessing abdominal CT-scans in a structured manner with signs specifically indicating internal herniation can considerably improve diagnostic accuracy [12–15]. However, the interobserver agreement for each of these signs varies considerably [12, 14, 16].

To date, no prediction score based solely on structured assessment of abdominal CT-scans is available [17]. Furthermore, it is unclear if signs specific for internal herniation are interpreted consistently by radiologists and radiology registrars with different levels of expertise. This study aims to develop an easily applicable and reproducible prediction score for internal herniation in patients presenting with abdominal pain following RYGB, based on CT-signs previously reported in literature.

## Materials and Methods

### Study Population

All patients presenting with abdominal pain more than 30 days post-RYGB were identified between January 2014 and December 2019 at a high-volume non-academic teaching hospital with a dedicated bariatric center. Inclusion required CT for suspected internal herniation followed by diagnostic laparoscopy during the same admission. All RYGB were performed exclusively by dedicated bariatric surgeons, followed the antecolic approach and mesenteric defects were always closed by hernia stapler (Endo UniversalTM 65, Covidien, USA). Diagnostic laparoscopy was always done by dedicated gastrointestinal or bariatric surgeons. This retrospective study was approved by the regional and institutional review board with informed consent waived.

### Reassessment, Definitions and Outcome Measures

CT-scans were coded, anonymized and were independently reassessed by two dedicated abdominal radiologists (experts), one senior radiology registrar (last year of training) and one junior radiology registrar (first year of training), blinded for any clinical information, other imaging and personal patient information. Based on literature, ten CT-signs associated with internal herniation were scored, including venous congestion, swirl sign, mesenteric edema, mushroom sign, clustered loops, enlarged nodes, hurricane eye sign, small bowel behind superior mesenteric artery (SMA), small bowel obstruction and right-sided anastomosis. Definitions of these signs have been previously reported [12, 18]. Further details were added for right-sided anastomosis (position of anastomosis with regard to the first descending part of the alimentary limb), venous congestion (tightened portal vein or its branches) and small bowel behind SMA (cranial of aortic bifurcation). A score was determined for suspicion for internal herniation and defined as the assessed likelihood an internal herniation was present on a 5-point Likert scale (score 5 being certain of presence of internal herniation, score 1 being certain no internal herniation is present). Readers received brief training on the CT-signs by an educational presentation of an experienced radiologist and by reading literature on the CT-signs.

Original reports were written predominantly by abdominal radiologists with some exceptions written by general radiologists and registrars. Scoring of the original interpretation of CT-scans was done by reading the original free text report of the CT-scan: when suspicion of internal herniation was documented, the original assessment was scored as ‘suspicion of internal herniation present’. Uncertainties during the scoring of the original assessment were discussed with one of the two aforementioned radiologists. Internal herniation was defined as being present or not, as documented in the surgical report. A laparoscopy negative for internal herniation was defined as closed internal spaces or nearly closed spaced with no evidence of internal herniation or obstructing pathology. Two researchers registered patient characteristics at time of RYGB (i.e. age, sex, weight, BMI, ASA) and characteristics at presentation with abdominal pain (weight, BMI, %TWL).

### Image Acquisition

CT-scans (Siemens/Philips, 1 mm slices) were acquired 70 s after administration of 80 mL intravenous contrast Xenetix 300 mg I/mL (Iobitridol, Guerbet, Gorinchem, the Netherlands) bolus intravenously at 4 mL/s. Multiplanar reconstructions were available.

### Statistical Analysis

Patient, presentation, and treatment-related characteristics are described as count with percentages, means with standard deviation (SD), or medians with interquartile range (IQR). Differences between groups were analyzed by Student’s *t* and Mann–Whitney *U* test (continuous data with a normal and skewed distribution). Categorical data were compared using Pearson chi-square or Fisher’s exact test. Missing values were excluded pairwise per variable.

Sensitivity, specificity, positive predictive value (PPV) and negative predictive value (NPV) were calculated using contingency tables for each CT-sign, original interpretation and radiological suspicion score. Univariable analysis was performed to identify associations between CT-signs and the presence of internal herniation and reported as relative risk ratios (RRs) with their respective 95% confidence intervals (CIs). Interobserver agreement was measured using Fleiss’ kappa, with *K* > 0.40 defined as adequate interobserver agreement. Differences in interobserver agreement between radiologists and registrars was analyzed with a 2-sample *Z* test.

Initial selection of predictors for model development included CT-signs previously described in literature and those with a *K* > 0.40 following interobserver analysis. To identify which CT-signs were independently associated with internal herniation, a multivariable logistic regression analysis with backward stepwise selection was performed based on Akaike’s information criterion. Bootstrapping with 200 resamples was performed to internally validate the diagnostic performance of the model. Based on the beta-regression coefficients of the retained predictive factors, a practical prediction score was developed. Calibration of the practical scoring system was assessed with a calibration plot.

The original assessment was compared to the structured assessment and prediction score model by performing receiver operating characteristics (ROC) curve analyses. Area under the curves (AUC) were computed and compared as measurement for diagnostic accuracy. Ideal cut-offs were determined by equally weighing sensitivity and specificity. Statistical analysis was performed using IBM SPSS Statistics version 29 (IBM corp., 2021), Rstudio (Rstudio Team 2023) and Python 3.8 (python Software foundation, 2019), with significance set at *P* < 0.05.

## Results

A total of 2773 patients underwent RYGB. At the time of screening (December 2022), 98 patients underwent 120 CT-scans followed by diagnostic laparoscopy in the same presentation of abdominal pain. Six scans were excluded due to poor quality or missing images, resulting in 92 patients with 114 scans (mean 1.24 scans/patient).

### Patient Demographics and Characteristics at Presentation with Abdominal Pain

Patient demographics and patient characteristics at the time of presentation with abdominal pain are found in Table 1. Most were female (83%), ASA 2 (71%), with mean age at the time of RYGB 45 years and BMI of 40.8 kg/m^2^. Median percentage of total weight loss (%TWL) was median 31.9% with mean BMI change of 13 kg/m^2^. Median time from RYGB until presentation was 711 days (IQR 372–1147). CT-scans were performed mostly on the day of presentation (IQR 0–1 days), with diagnostic laparoscopy within a median of 1 day after CT-scan (IQR 0–6.5 days).

**Table 1:** Patient demographics and characteristics at presentation with abdominal pain

	Total patients *N* = 92	Total CT’s *N* = 114	No internal herniation *N* = 70	Internal herniation *N* = 44	*P*-value
Male Female	16 (17) 76 (83)				
Age at RYGB, years	45 ± 9				
ASA 1 2 3	4 (4) 65 (71) 23 (25)				
Weight RYGB, KG	118 ± 18				
BMI RYGB, kg/m^2^	40.8 ± 4.4				
Weight presentation, kg *		80 ± 15	79 ± 14	82 ± 17	0.359
BMI presentation, kg/m^2^ *		27.6 ± 4.6	27.6 ± 4.2	27.7 ± 5.2	0.846
BMI change *		13 ± 5.2	13.0 ± 5.1	12.9 ± 5.4	0.899
%TWL *		31.9 (25.7–40.1)	32.0 (26.3–39.5)	30.8 (25.2–42.3)	0.941
Time RYGB until presentation, days		711 (372–1147)	843 (467–1207)	553 (285–966)	0.014
Time presentation until CT-scan, days		0 (0–1)	0 (0–1)	0 (0–1)	0.598
Time CT-scan until DLS, days		1 (0–6.5)	1 (0–34)	1 (0–2.75)	0.160

Values are presented as number (%) and mean ± standard deviation or median (IQR 25–75%)

*RYGB* laparoscopic Roux-en-Y gastric bypass, *BMI* body mass index, *%TWL* percentage of total weight loss

Missing values were excluded pairwise

^*^Weight *n* = 110, BMI change was defined as ∆BMI: BMI at RYGB—BMI at presentation

Diagnostic laparoscopy identified 44 cases of internal herniation (24 Petersen’s space, 18 jejunojejunostomy site, one both defects, one unknown). Patients with internal herniation presented earlier with abdominal pain after RYGB than patients without internal herniation (*p* = 0.014, 553 days to 843 days). No significant differences were found with regard to weight, weight loss at time of presentation, timing of CT-scan and diagnostic laparoscopy.

### Diagnostic Accuracy of Original Interpretation and Overall Suspicion After Structured Assessment

ROC analysis (Table 2) showed AUCs of 0.69 (original interpretation) versus 0.71–0.79 (systematic assessment by all readers). Radiologist 1 scored an AUC of 0.79 for overall suspicion (score 1–4 versus score 5), significantly higher than the original interpretation. A score of 1–4 versus score 5 showed the highest AUC and had the least variability in score between the different readers.

**Table 2:** Receiver operating characteristics analysis estimates for internal herniation according to the original interpretation, systematic CT assessment and the prediction model

	AUC (95% CI)	Cut-off	SE (%)	SP (%)	PPV (%)	NPV (%)	*P*-value
Original CT interpretation	0.69 (0.59–0.80)	No IH vs. definite IH	54 (24/44)	85 (59/70)	67 (24/35)	75 (59/79)	Ref
Systematic assessment							
*Radiologist 1*	0.79 (0.69–0.88)	Likert 5 versus 1–4	68 (30/44)	90 (63/70)	81 (30/37)	82 (63/77)	0.030
*Radiologist 2*	0.77 (0.67–0.87)	Likert 5 versus 1–4	61 (27/44)	93 (65/70)	84 (27/32)	79 (65/82)	0.096
*Senior registrar*	0.76 (0.67–0.86)	Likert 5 versus 1–4	68 (30/44)	84 (59/70)	73 (30/41)	81 (59/73)	0.387
*Junior registrar*	0.71 (0.60–0.81)	Likert 5 versus 1–4	50 (22/44)	91 (64/70)	79 (22/28)	74 (64/86)	0.980
Prediction model	0.79 (0.70–0.88)	Score ≥ 2 vs. score < 2	75 (33/44)	71 (50/70)	62 (33/53)	82 (50/61)	0.038

*AUC* area under the curve, *SE* sensitivity, *SP* specificity, *PPV* positive predictive value, *NPV* negative predictive value

Radiologist = dedicated abdomen radiologist, registrar = in training for radiologist

*P*-value based on comparison of difference in AUC

### Diagnostic Accuracy of Specific CT-Signs for Internal Herniation

Since radiologist 1 showed the highest diagnostic accuracy (AUC) for overall suspicion score, further analysis used their scores. Diagnostic accuracy per individual sign for the other readers can be found in the Supplementary Material (Table S1). Univariable logistic regression analysis of CT-signs is shown in Table 3. Every individual sign was significantly scored more often in the group with internal herniation versus without internal herniation. PPV was highest for swirl sign (81%), hurricane eye sign (81%) and small bowel behind SMA (78%). NPV was best for venous congestion (81%), mushroom sign (80%) and mesenteric edema (78%). Venous congestion (RR 3.7; 2.2–6.2), mushroom sign (RR 3.4; 2.0–5.7), swirl sign (RR 2.8; 1.9–4.1) and mesenteric edema (RR 2.8; 1.7–4.5) scored the highest relative risk ratios for internal herniation.

**Table 3:** Univariable logistic regression analysis of specific CT findings in relation to internal herniation

CT-sign	No internal herniation (*N* = 70)	Internal herniation (*N* = 44)	Positive predictive value	Negative predictive value	RR (95% CI)	*P* value
Venous congestion	14 (20)	31 (70)	31/45 (69)	56/69 (81)	3.7 (2.2–6.2)	< 0.001
Swirl sign	4 (6)	17 (39)	17/21 (81)	66/93 (71)	2.8 (1.9–4.1)	< 0.001
Mesenteric edema	18 (26)	29 (66)	29/47 (62)	52/67 (78)	2.8 (1.7–4.5)	< 0.001
Mushroom sign	14 (20)	30 (68)	30/44 (68)	56/70 (80)	3.4 (2.0–5.7)	< 0.001
Clustered loops	15 (21)	24 (54)	24/39 (62)	55/75 (73)	2.3 (1.5–3.6)	< 0.001
Enlarged nodes	9 (13)	17 (39)	17/26 (65)	61/88 (69)	2.1 (1.4–3.2)	< 0.001
Hurricane eye sign	3 (4)	13 (30)	13/16 (81)	67/98 (68)	2.6 (1.8–3.7)	< 0.001
Small bowel behind SMA	2 (3)	7 (16)	7/9 (78)	68/105 (65)	2.2 (1.4–3.4)	< 0.001
Small bowel obstruction	6 (9)	11 (25)	11/17 (65)	64/97 (66)	1.9 (1.2–3.0)	< 0.001
Right-sided anastomosis	10 (14)	22 (50)	22/32 (69)	60/82 (73)	2.6 (1.7–3.9)	< 0.001

Data presented as counts with percentages in the parentheses. Data based on radiologist 1

*RR* risk ratio, *CI* confidence interval, *SMA* superior mesenteric artery

### Interobserver Agreement on Individual CT-Signs and Overall Suspicion Scores

Interobserver agreement between all readers was good for overall suspicion score, venous congestion, swirl sign, mesenteric edema, mushroom sign, small bowel obstruction and right-sided anastomosis (Table 4). Agreement between the experts was good for almost all CT-signs, except for clustered loops (*K* = 0.08) and hurricane eye sign (*K* = 0.40). Significant differences between experts and registrars were seen for five-point suspicion score, venous congestion and enlarged.

**Table 4:** Interobserver agreement on the CT-signs for internal herniation

Score/CT-sign	All readers (*N* = 4)	Experts (*N* = 2)	Registrars (*N* = 2)	*P* value*
Binary scale score 5 vs. score 1–4	0.78 (0.70–0.85)	0.81 (0.63–0.99)	0.73 (0.54–0.91)	0.548
Binary scale score 4–5 vs. score 1–3	0.75 (0.68–0.83)	0.94 (0.76–1.0)	0.56 (0.38–0.75)	0.004
Five-point scale	0.40 (0.36–0.45)	0.58 (0.46–0.69)	0.18 (0.07–0. 28)	< 0.001
Venous congestion	0.75 (0.67–0.83)	0.87 (0.69–1.0)	0.58 (0.4–0.76)	0.029
Swirl sign	0.65 (0.57–0.72)	0.65 (0.46–0.83)	0.63 (0.45–0.82)	0.916
Mesenteric edema	0.64 (0.57–0.71)	0.69 (0.51–0.88)	0.59 (0.41–0.79)	0.332
Mushroom sign	0.63 (0.56–0.71)	0.56 (0.38–0.74)	0.64 (0.46–0.82)	0.547
Clustered loops	0.20 (0.12–0.28)	0.08 (0–0.26)	0.151 (0–0.37)	0.596
Enlarged nodes	0.40 (0.32–0.48)	0.61 (0.43–0.80)	0.31 (0.13–0.50)	0.024
Hurricane eye sign	0.32 (0.24–0.39)	0.40 (0.21–0.58)	0.19 (0.01–0.38)	0.116
Small bowel behind SMA	0.31 (0.23–0.38)	0.50 (0.31–0.68)	0.27 (0.08–0.45)	0.085
Small bowel obstruction	0.60 (0.52–0.67)	0.77 (0.59–0.96)	0.58 (0.4–0.77)	0.156
Right-sided anastomosis	0.56 (0.49–0.64)	0.64 (0.46–0.83)	0.44 (0.25–0.62)	0.074

Data presented as *K* values with 95% confidence interval in parentheses

*SMA* superior mesenteric artery

Experts = dedicated abdomen radiologists, registrar = in training for radiologist

^*^P value for difference between experts and registrars

### Prediction Scoring Model for Internal Herniation Based on CT-Signs

CT-signs with interobserver agreement of *K* > 0.40 between all readers (*n* = 6) were included in multivariable logistic regression (Table S2). After backward selection, three signs remained in the final model as the strongest predictors for internal herniation: venous congestion (OR 4.41, *p* = 0.005) swirl sign (OR 3.23, *p* = 0.090) and right-sided anastomosis (OR 2.47, *p* = 0.093). A practical score was developed, assigning 1 point to swirl sign and right-sided anastomosis, and 2 points to venous congestion.

**Fig. 1:**
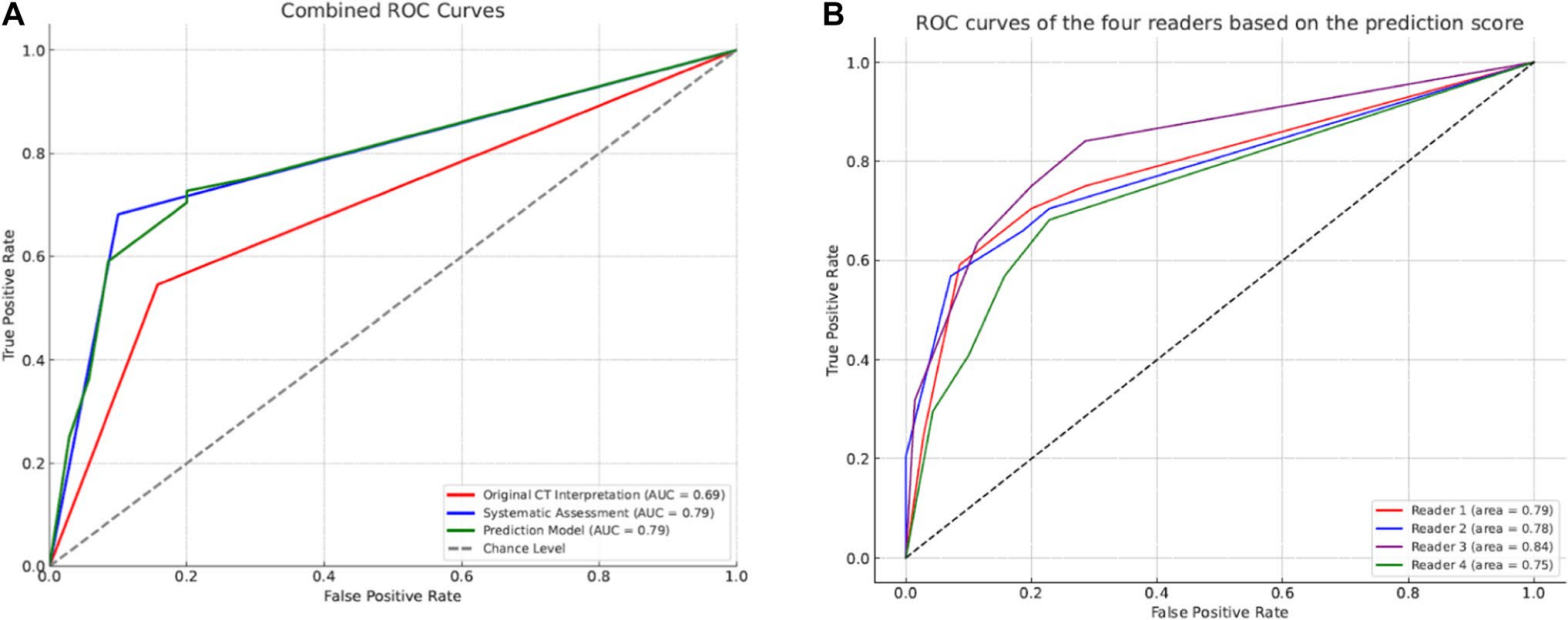
Receiver operator curves combined and for prediction score on data of each reader. **A** ROC curve analysis of the prediction model, systematic assessment by best scoring radiologist 1 and original CT interpretation, indicating their ability to discriminate between patients with and without internal herniation. **B** ROC curve analysis of the prediction model applied to data of each individual reader. Reader 1 and reader 2 are dedicated abdomen radiologists, reader 3 is the senior registrar, reader 4 is the junior registrar

The optimal cut-off point was identified on a total of 2 points (ROC analysis), in which patients with scores ≥ 2 points were considered at high risk of internal herniation. The cut-off value of ≥ 2 yielded a sensitivity of 75% and specificity of 71%, and AUC of 0.79 based on data from radiologist 1 (Table 2). Figure 1A summarizes the ROC curves of the original interpretation, the systematic assessment of radiologist 1 and the prediction score applied to data of radiologist 1. Applying the score model to the other three readers demonstrated comparable or higher AUCs compared to their systematic assessment (Fig. 1B).

The diagnostic performance for each score of the three-sign model for identifying internal herniation is given in the Supplemental Material (Table S3). Internal validation with bootstrapping demonstrated an AUC of 0.78. The calibration plot demonstrated adequate calibration of the model.

## Discussion

In this study, we developed an easily applicable prediction score for internal herniation based on literature-identified CT-signs in patients presenting with abdominal pain post-RYGB. This three-sign score (venous congestion 2 points, swirl sign and right-sided anastomosis both 1 point), significantly improves diagnostic accuracy compared to the original assessment. Notably, when the score was applied to all readers individually, all AUCs demonstrated comparable or even higher diagnostic accuracy compared to their structured assessment.

The importance of structured assessment of CT-scans for detection of internal herniation has previously been shown by other studies [6, 12, 19, 20]. Ederveen et al. showed an increase of 55.6% to 81.3% in PPV and 95.8% to 96% in NPV when comparing free text reporting to structured assessment [12]. Our study re-enforces these finding demonstrating a significant improvement in diagnostic accuracy (AUC of 0.79), with PPV improving to 81% and NPV to 82%. Given that all ten previously described CT-signs were individually and significantly more prevalent in patients with internal herniation—confirming previous studies—, it is compelling to use these signs in clinical practice [7, 8, 12, 20]. However, the assessment of these ten signs can be a tedious task, and their interobserver reproducibility is not well-documented. Our model not only aligns with the improvements found in the previous studies but also offers a more streamlined, reproducible approach.

In the current study, interobserver agreement between all readers was good for overall suspicion and for most individual signs, confirming previous research [13, 15, 20]. This indicates that with training it is possible for radiologists and registrars to learn how to systematically assess CT-scans post-RYGB. However, some signs are interpreted notably different even by experienced radiologists (clustered loops and hurricane eye sign in our study), indicating that their definitions might be unclear and questioning their added value to structured assessment. Our prediction score therefore includes only reproducible CT-signs with adequate interobserver agreement offering a simplified and reproducible alternative to extensive assessment, without compromising the improved diagnostic effectiveness in experienced radiologists. Furthermore, applying the prediction score, diagnostic accuracy improved for less experienced registrars too, introducing a reliable aid for structured assessment of CT-scans for internal herniation.

A previously reported prediction model including both clinical parameters (EWL > 95%) and CT signs showed adequate discrimination and an AUC of 0.799 [6]. Although it is known that more weight loss increases the risk of internal herniation [15, 19, 21], hypothetically through loss of mesenteric fat, the vast majority of patients do not reach an EWL of 95% or higher [6, 17]. Alternatively, internal herniation also occurs in patients with an EWL lower than 95%, ranging from EWL 59 to 75% [19, 21]. In our results, %TWL was not significantly higher in the group with internal herniation and internal herniation. A possible explanation could be that patients with internal herniation presented earlier after RYGB than the patients without internal herniation. Our prediction model focused solely on CT-signs to avoid including parameters that could differ between populations, and to make a clean, easily applicable score that could be used for every patient presenting with abdominal pain and a suspicion for internal herniation.

Some limitations apply to our study. We choose to score all scans by CT-signs previously described in current literature, which may have resulted in the omission of less known signs. Nevertheless, the diagnostic accuracy of the used signs is well documented, and our study and model further demonstrate their added clinical value. The retrospective nature of the study introduces a partial verification bias, which could potentially result in an overestimation of the sensitivity by missing false negative CT-scans when diagnostic laparoscopy is not performed subsequently. To reduce this risk, we made sure we also included every patient who underwent diagnostic laparoscopy in the follow-up when initial conservative treatment failed. Furthermore, although our prediction score is internally validated, a next step would be to validate externally to account for different population characteristics and use of different surgical techniques [22]. Possibly some signs could be more predictive for internal herniation through Petersen’s or the jejunojejunostomy site. We investigated this hypothesis in our dataset but no sign scored a hundred percent presence for one or the other. For the purpose of this study, we believe our prediction score adequately aids in estimating the risk of internal herniation in general and therefore supports the decision-making process for performing a diagnostic laparoscopy. At last, in this study, an original free-text report was written by different readers. Results could therefore reflect the difference of these readers to our dedicated abdominal radiologists rather than the difference in free text versus structured assessment. Nonetheless, ROC analysis showed improvement of AUC’s comparing free text to structured assessment for every individual reader, even the senior and junior registrars, suggesting that the bias is minimal. And most importantly, the prediction model showed even more improvement when applying the model to the scored CT-signs for the individual readers compared to their own structured assessment.

At the moment, the yield of diagnostic laparoscopy for internal herniation ranges from 50–70% [7–10, 23, 24]. Correctly choosing for conservative treatment will not only save patients unnecessary surgery with all additional risks, it will also decrease the burden on the healthcare system and costs. Our prediction score is reliable and easily applicable in clinical practice. It takes into account the subjective nature of assessments by only using signs that are reproducible by readers of different levels of expertise. Although the optimal cut-off for diagnostic accuracy is ≥ 2 points, the risk score table (Supplemental Material, Table S3) allows for clinicians to decide at which score a diagnostic laparoscopy is indicated and when observation is permissible.

In conclusion, our prediction-model with three signs offers a simplified and reproducible alternative to extensive assessment, without compromising the improved diagnostic effectiveness. This prediction score could provide a useful tool in clinical practice to aid in the decision-making process to perform a diagnostic laparoscopy for suspicion of internal herniation. Since more prediction models for internal herniation are recently published, future studies should focus on integrating all these models into one optimal diagnostic algorithm for clinical practice.

## Supplementary Information

Below is the link to the electronic supplementary material.

Supplementary Material 1 (DOCX 23.5 KB)

## Data Availability

No datasets were generated or analysed during the current study.
