# Supplementary material for: Optimal Use of Computed Tomography in Diagnosing Internal Herniation After Roux-en-Y Gastric Bypass: A Proposition for the Application of a Radiological Prediction Score
Source: Obes Surg. 2025 Oct 18;35(12):5136–44. doi: 10.1007/s11695-025-08323-4 (PMC12722339; doi:10.1007/s11695-025-08323-4)
Supplement: Supplementary file 1 — Supplementary Material 1 (DOCX 23.5 KB) [file 11695_2025_8323_MOESM1_ESM.docx]

**Supplementary tables and figures**

*Table S1. Diagnostic accuracy CT-signs per reader*

|  | Radiologist 2 | | senior registrar | | Junior registrar | |
| --- | --- | --- | --- | --- | --- | --- |
| CT-sign | Sensitivity (n=44) | Specificity (n=70) | Sensitivity (n=44) | Specificity (n=70) | Sensitivity (n=44) | Specificity (n=70) |
| Venous congestion | 66 (29) | 81 (57) | 75 (33) | 80 (55) | 55 (24) | 84 (59) |
| Swirl sign | 32 (14) | 97 (68) | 55 (24) | 97 (68) | 41 (18) | 87 (61) |
| Mesenteric edema | 66 (29) | 73 (51) | 77 (34) | 51 (36) | 66 (29) | 69 (48) |
| Mushroom sign | 48 (21) | 94 (66) | 36 (16) | 91 (64) | 55 (24) | 87 (61) |
| Clustered loops | 25 (11) | 94 (66) | 66 (29) | 60 (42) | 50 (22) | 74 (51) |
| Enlarged nodes | 25 (11) | 94 (66) | 46 (20) | 59 (41) | 25 (11) | 87 (61) |
| Hurricane eye sign | 11 (5) | 99 (69) | 36 (16) | 81 (57) | 16 (7) | 99 (69) |
| Small bowel behind SMA | 18 (8) | 93 (65) | 41 (18) | 74 (52) | 14 (6) | 87 (61) |
| Small bowel obstruction | 16 (7) | 91 (64) | 36 (16) | 76 (53) | 34 (15) | 86 (60) |
| Right-sided anastomosis | 50 (22) | 91 (64) | 50 (22) | 81 (57) | 46 (20) | 91 (64) |

*Data presented as percentages (count). SMA = superior mesenteric artery.*

*Table S2. Multivariable logistic regression analysis of CT findings significantly associated with internal herniation in univariable analysis and a K-statistic >0.40*

| CT-sign | *β* regression coefficient | OR (95% CI) | *p* value | Points^†^ |
| --- | --- | --- | --- | --- |
| Venous congestion | 1.49 | 4.41 (1.56-12.50) | 0.005 | 2 |
| Swirl sign | 1.17 | 3.23 (0.83-12.53) | 0.090 | 1 |
| Right-sided anastomosis | 0.91 | 2.47 (0.86-7.10) | 0.093 | 1 |

*OR = odds ratio, CI = confidence interval
†Assignment of points to CT findings was based the corresponding β regression coefficient. Scaling was performed with respect to the discriminatory power of the scores as determined by ROC analysis.
Intercept β = -1.60*

*Table S3. Risk scores and their coordinates on the ROC curve*

| Risk score | n | Observed IH risk | Sensitivity (%) | Specificity (%) | PPV (%) | NPV (%) |  |
| --- | --- | --- | --- | --- | --- | --- | --- |
| *Internal herniation score* | | | | | | | |
| score 0 | 61 | 0.18 | 100 | 0 | 39 | 0 |  |
| score 1 | 8 | 0.25 | 75 | 71 | 62 | 82 |  |
| score 2 | 13 | 0.38 | 71 | 80 | 69 | 81 |  |
| score 3 | 19 | 0.79 | 59 | 91 | 81 | 78 |  |
| score 4 | 13 | 0.85 | 25 | 97 | 85 | 67 |  |

*ROC = receiver operating characteristics. IH = internal herniation. Sensitivity, specificity, positive predictive value and negative predictive value defined by their coordinates on the ROC curve*
